# Single-Molecule Investigation of the Binding Interface Stability of SARS-CoV-2 Variants with ACE2

**DOI:** 10.1021/acsnanoscienceau.3c00060

**Published:** 2024-03-08

**Authors:** Ankita Ray, Thu Thi Minh Tran, Rita dos Santos Natividade, Rodrigo A. Moreira, Joshua D. Simpson, Danahe Mohammed, Melanie Koehler, Simon J. L Petitjean, Qingrong Zhang, Fabrice Bureau, Laurent Gillet, Adolfo B. Poma, David Alsteens

**Affiliations:** † Louvain Institute of Biomolecular Science and Technology, Université catholique de Louvain, 1348 Louvain-la-Neuve, Belgium; ‡ Faculty of Materials Science and Technology, University of Science—VNU HCM, 227 Nguyen Van Cu Street, District 5, 700000 Ho Chi Minh City, Vietnam; § Vietnam National University, 700000 Ho Chi Minh City, Vietnam; ∥ Basque Center for Applied Mathematics, Mazarredo 14, 48009 Bilbao, Spain; ⊥ Laboratory of Cellular and Molecular Immunology, GIGA Institute, Liège University, 4000 Liège, Belgium; # Immunology-Vaccinology Lab of the Faculty of Veterinary Medicine, Liège University, 4000 Liège, Belgium; ∇ Institute of Fundamental Technological Research, Polish Academy of Sciences, Pawińskiego 5B, 02-106 Warsaw, Poland; ○ WELBIO department, WEL Research Institute, 1300 Wavre, Belgium

**Keywords:** SARS-CoV-2, ACE2, RBD, Atomic force microscopy, Steered molecular dynamics, biolayer interferometry, convalescent patient sera

## Abstract

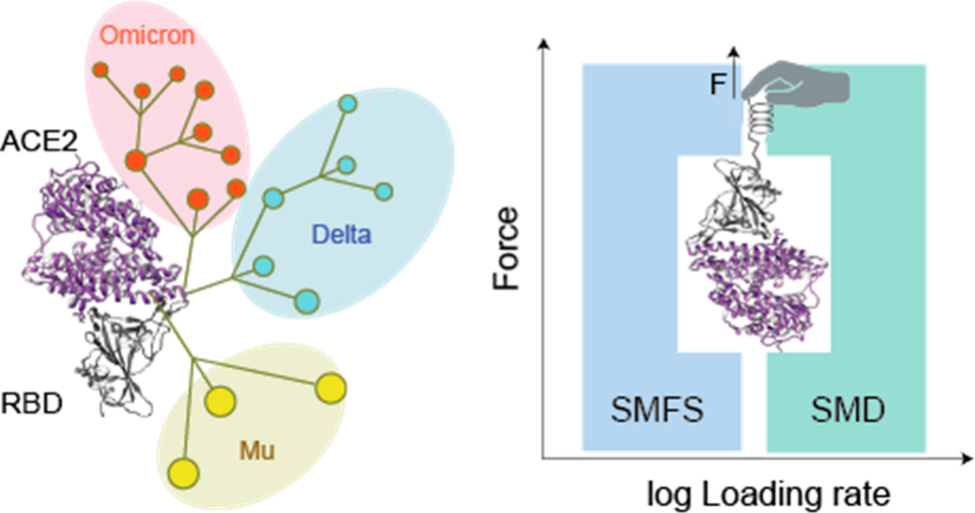

The SARS-CoV-2 pandemic spurred numerous research endeavors to comprehend the virus and mitigate its global severity. Understanding the binding interface between the virus and human receptors is pivotal to these efforts and paramount to curbing infection and transmission. Here we employ atomic force microscopy and steered molecular dynamics simulation to explore SARS-CoV-2 receptor binding domain (RBD) variants and angiotensin-converting enzyme 2 (ACE2), examining the impact of mutations at key residues upon binding affinity. Our results show that the Omicron and Delta variants possess strengthened binding affinity in comparison to the Mu variant. Further, using sera from individuals either vaccinated or with acquired immunity following Delta strain infection, we assess the impact of immunity upon variant RBD/ACE2 complex formation. Single-molecule force spectroscopy analysis suggests that vaccination before infection may provide stronger protection across variants. These results underscore the need to monitor antigenic changes in order to continue developing innovative and effective SARS-CoV-2 abrogation strategies.

## Introduction

**Figure 1:**
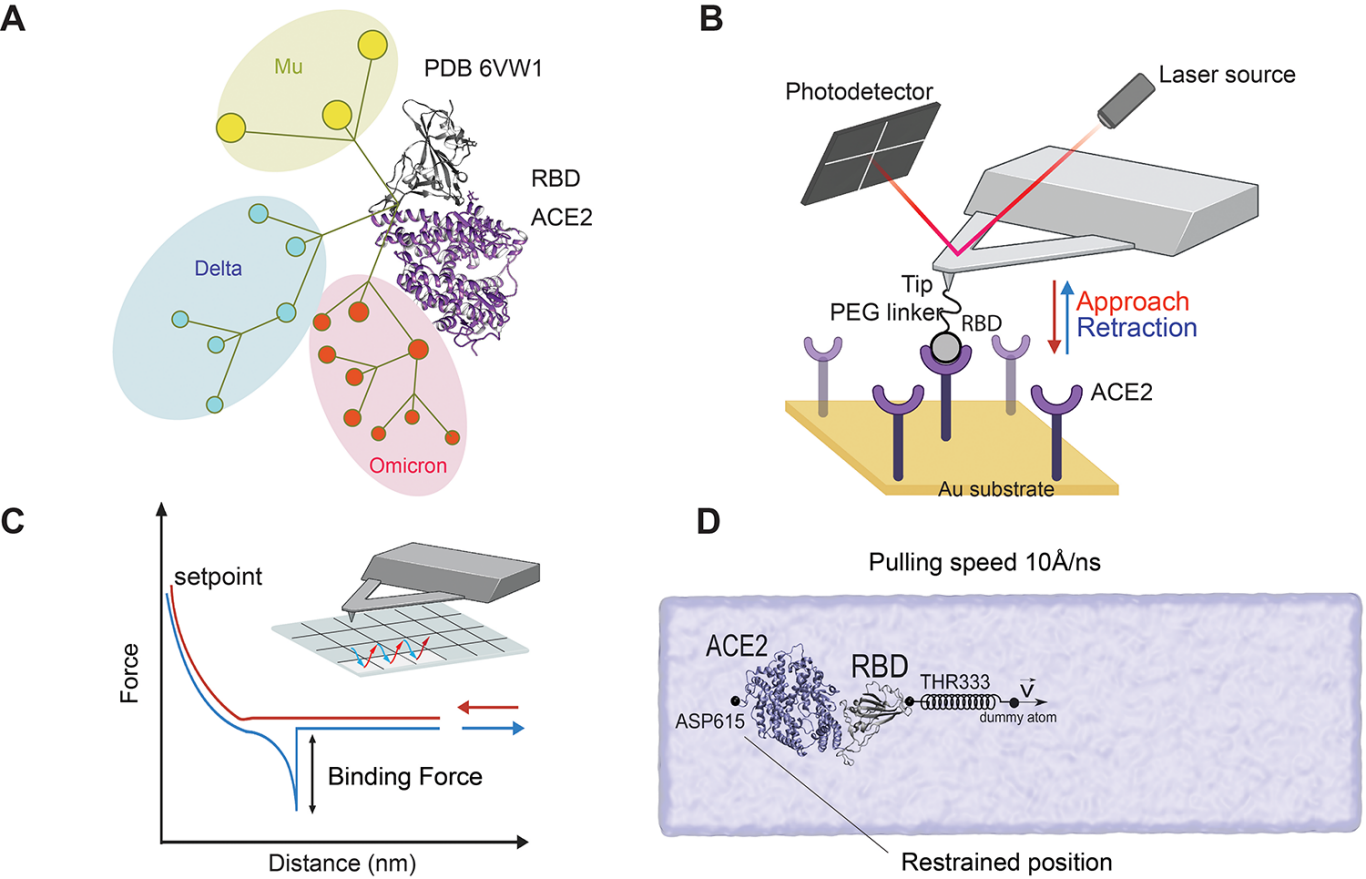
Single-molecule investigation of SARS-CoV-2 variants using AFM and SMD simulation. (A) Phylogenetic tree of SARS-CoV-2 showing the emergence of the variants of concern (VoCs) Omicron, Delta, and Mu. (B) Probing of RBD mutants binding to ACE2 receptors using atomic force microscopy (AFM). (C) AFM tip probes the interaction during the pixel-by-pixel scanning of the sample and extracts from each pixel a force–distance (FD) curve obtained by making cycles of approach and retraction. (D) All-atom steered molecular dynamics (SMD) simulation with the tethered ACE2 protein showing the effect of force pulling on the RBD protein. The complex is placed in a cubic solvent box containing 0.15 mol L^–1^ of NaCl molecules.

The first outbreak of severe acute respiratory syndrome (SARS) in 2003 and the recent outbreak of SARS coronavirus 2 (SARS-CoV-2) show very different patterns of transmissibility and pathogenicity. While the former remained highly localized, SARS-CoV-2 resulted in a global pandemic. Soon, the Wuhan strain evolved into numerous variants of concern (VoCs), demonstrating how easily this virus acquires mutations in its spike (S) glycoprotein without loss of fitness.^1−3^ Although mutations were anticipated, the first VoCs to emerge primarily possessed mutations in the binding interface between the S glycoprotein and angiotensin-converting enzyme 2 (ACE2) receptor (Figure 1A).^2,4−7^ The main effect of these mutations was to increase the stability of the binding complex. For example, N501Y, which is present in all VoCs except Delta, is thought to increase the level of ACE2 binding.^7,8^ However, with the appearance of the first immunized people (either through vaccination or previous contact with the virus), the virus came under enormous selection pressure and began to mutate even more rapidly.^3,6,9−11^

Specific mutations in the S glycoprotein increase its fitness, thereby dramatically altering its antigenic landscape. For instance, present in all VoCs, D614G is responsible for large conformational changes that facilitate the transition from the closed to open state of the receptor-binding domain (RBD) improving ACE2 binding.^12−15^ Other mutations localized in the receptor binding motif (RBM) influence bond stability with ACE2 receptor and affect the efficiency of monoclonal antibody (mAb) neutralization.^16^ This is largely due to the RBM being a prime target for neutralizing antibodies induced by infection or current vaccines.^17^ As illustrated by the E484K mutation in Beta and Gamma VoCs that led to the inefficacy of previously developed monoclonal antibodies approved as treatment for COVID-19.^10^ In addition to the RBD, the N-terminal domain (NTD), which interacts with auxiliary receptors including DC-SIGN/L-SIGN,^18^ is another major target of neutralizing antibodies. However, serological analysis of plasma from individuals infected with SARS-CoV-2 revealed that ∼65%–80% of circulating antibodies target the RBD, while only ∼6%–20% target the NTD, with the remainder targeting the S2 subunit.^19^ The numerous mutations observed in the RBD and their effects on glycosylation and glycan shielding are not fully understood, although it has been shown that all mutations affect spike behavior at several levels: changes in glycosylation profile,^20^ changes in receptor binding,^21,22^ altered spike stability^23^ and antibody recognition.^24^

In this regard, a better understanding of the impact of spike protein mutations on the spike-receptor interaction, as well as their influence on inhibition by antibodies and immune responses at atomic resolution, is of critical importance. For this application, atomic force microscopy (AFM) has been proven to be a highly sensitive technique,^25,26^ being able to measure nanoscale forces between the S-glycoprotein and the ACE2 receptors on model surfaces and living cells.^18,22,27^ In addition, previous AFM experiments in combination with molecular dynamics (MD) simulation have enabled us to map the binding interface, thereby enhancing our biophysical understanding of the complex between the S glycoprotein and ACE2 receptor and moreover allowing examination of structural correlation with antigenicity.^27,28^

In this study, we deciphered the RBD/ACE2 dissociation process of newer VoCs under a mechanical load (Figure 1B–D). We calculated the mechanical strength, kinetic and thermodynamic parameters describing the binding free energy landscape of the complex. This highlights the evolution of the binding interface that results in altered stability with its cognate receptor and recognition by neutralizing antibodies, the latter potentially leading to escape from humoral immunity induced by prior infection or vaccination.

## Results and Discussion

### Probing VoCs RBD/ACE2 Binding Free-Energy Landscape

**Figure 2:**
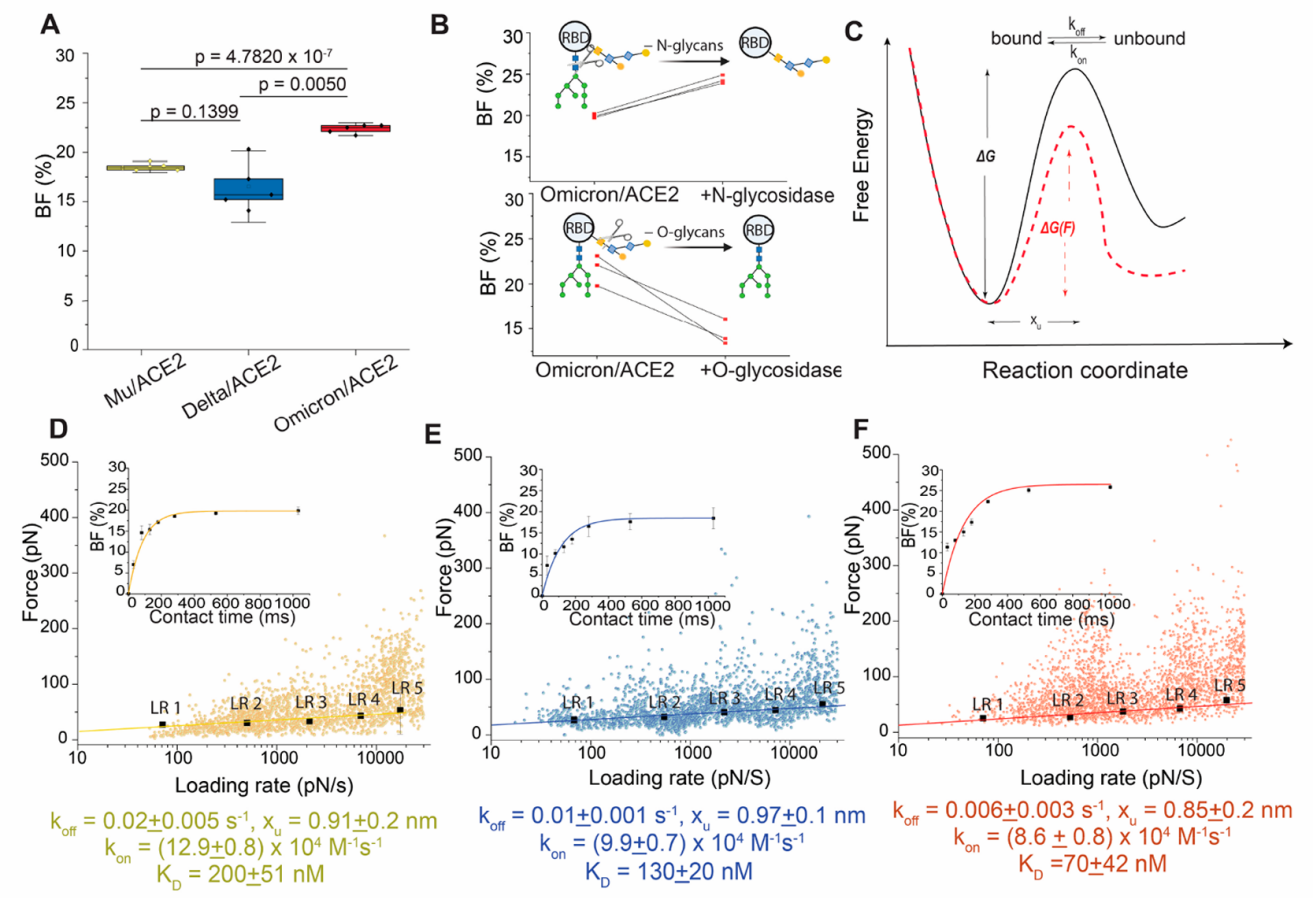
Probing the binding free-energy landscape of the RBD/ACE2 complexes by AFM. (A) Box-whiskers plot of the binding frequencies (BF) measured by AFM between the functionalized tip (RBD mutants) and the grafted ACE2 model surface. Each data point corresponds to a map of 1024 FD curves measured at an approach and retract speed of 1 μm/s and a dwell time of 250 ms. (B) Before-after plots of specific BFs showing the effect of deglycosylation after enzymatic treatment of the functionalized cantilever with *N*-glycosidase (top) and *O*-glycosidase (bottom), respectively. One data point belongs to the BF from one map acquired at 1 μm/s retraction speed. (C) Bell–Evans (BE) model describing a ligand–receptor bond as a simple two-state model. The bound state is separated from the unbound state by a single energy barrier located at distance *x*_u_. The rupture force required to break a noncovalent bond follows a probabilistic distribution related to the energy landscape of the bond, describing how the probability of bond rupture increases exponentially with applied force. Experimentally, *k*_off_ can be estimated by probing the binding strength of a molecular complex under increasingly applied loads Lowering of the activation energy upon application of an external force (*F* > 0) is shown as red dotted lines. *k*_off_ and *k*_on_ represent the dissociation and association rate, respectively. (D–F) Dynamic force spectroscopy (DFS) plot showing the force extracted from individual FD curves (colored dots) as well as the mean rupture forces, determined at various loading rate (LR) ranges measured either between ACE2 receptor and Mu-RBD (D, *N* = 2785 data points), Delta-RBD (E, *N* = 2411 data points), and Omicron-RBD (F, *N* = 2698 data points). Data corresponding to single interactions were fitted with the BE model (straight line), providing average *k*_off_ and *x*_u_ values. Plots in the inset: BF (expressed in percentage) plotted as a function of the contact time.

In order to study the stability of RBD/ACE2 binding interfaces of three different VoCs (Mu, Delta, and Omicron), we first force probed the respective RBD/ACE2 complexes by AFM, using single-molecule force spectroscopy (SMFS). To probe the stability of the complex, surfaces were covalently grafted with ACE2, as previously described.^27^ The RBDs were tethered onto the tips at the end of a heterobifunctional polyethylene glycol (PEG) spacer, providing sufficient conformational mobility for the RBD to establish a stable complex with the ACE2 molecules. We selected covalent chemistry to immobilize ACE2 on a surface and RBD on an AFM tip, ensuring the specific detection of binding complex rupture. Additionally, molecules produced in human cell lines with post-translational glycosylation were selected to best preserve native shielding, domain binding accessibility, and conformational change dynamics critical for our study. Through repeated approach and retraction cycles, we extracted the binding frequencies (BF) for various RBDs. Binding events on the FD curves were considered to be specific if they (i) were significantly separated from the baseline noise (at least a 3-fold difference), (ii) were located at a distance >12 nm from the contact point (consistent with the PEG spacer extension), and (iii) were consistent with the extension of a protein-based polymer (fitted with the worm-like chain model). Notably, Omicron-RBD has a significantly higher BF compared to that of Mu and Delta (Figure 2A). We further investigated the impact of glycans to understand the molecular basis of overall antigenicity. Glycans are complex sugar molecules that are commonly found attached to proteins and lipids on the surface of cells, including viruses, and play a crucial role in various biological processes, including viral infection and immune recognition. Glycans on the surface of SARS-CoV-2 are involved in interactions with host cells and the immune system. Understanding these glycans can help in the development of vaccines and therapies.^18,29,30^ To understand the effect of glycan shielding upon the formation of the Omicron RBD/ACE2 binding complex, we measured and compared the BF before and after treatment with *N*- and *O*-glycosidases. Our findings revealed that the removal of N-linked glycans led to an increase in BF, whereas removal of O-glycans slightly reduced it (Figures 2B and S1B). This observation underscores the pivotal role of N-glycans in shielding and emphasizes the importance of investigating glycosylated receptors when studying the kinetics of binding complex formation. This behavior highlights the importance of studying the binding complex stability of variant RBD/ACE2, as it provides insights into underlying molecular interactions and potentially informs development of targeted therapeutic strategies as SARS-CoV-2 continues to mutate. Furthermore, to prove the specificity of this interaction, we measured the BF between RBD-functionalized cantilever and the gold surface lacking the hACE2 receptor and found significantly lower BF (<3%), corresponding to a nonspecific interaction (Figure S1B).

To understand the dynamics of these complexes, we sought to characterize their underlying kinetics and thermodynamics. The interaction can be described as an energy landscape with two states separated by an activation energy located at a distance of x_u_. The height of this barrier influences the kinetic association (*k*_on_) and dissociation rate (*k*_off_) (Figure 2C). To achieve this, the AFM tip is retracted at different speeds, resulting in various loading rates (LRs) (Figures S2–S4). The rupture forces are then plotted against the LR on a semilog graph scale, called dynamic force spectroscopy (DFS) plots (Figure 2D–F).

Overall, we observed that RBD/ACE2 complexes withstood forces in the range of 20–500 pN for all three VoCs (Figure 2D–F). Since the Bell-Evans model predicts that the rupture force of a bond is directly proportional to the logarithm of the LR, these forces need to be analyzed for small ranges of LR. Therefore, the bond strengths were sorted for smaller LR ranges and displayed as histograms (Figures S2–S4). These histograms show multiple peaks, confirming the presence of both single bond breakage and the simultaneous breakage of multiple bonds (also called multivalent bonds, resulting from the simultaneous interaction between multiple RBDs attached to the AFM tip and multiple ACE2s on the surface).^22,27^ The different histograms were fitted with multiple Gaussian fits, allowing us to extract the mean rupture force for a single bond rupture. These means are then superimposed on the respective DFS plots and fitted by a linear regression. Based on this model, the *x*_u_ and *k*_off_ parameters were then extracted from the slope and the intercept of the fit extrapolated to zero force, respectively.^31−33^ Based on this analysis, we obtained *x*_u_ of 0.91 ± 0.20 and 0.97 ± 0.10 nm for Mu and Delta, respectively, and a slightly lower value of 0.85 ± 0.20 nm for Omicron (Figure 2D-F). While the values for Mu and Delta are similar to the value previously observed for the RBD^WT^/ACE2 interface, the slightly lower value observed for Omicron could suggest lower flexibility of the binding interface. A decrease in the dissociation rate was obtained in the following order: Mu-RBD (0.026 ± 0.005 s^–1^) > Delta-RBD (0.013 ± 0.001 s^–1^) > Omicron-RBD (0.006 ± 0.003 s^–1^), with Omicron forming a 2-fold and 3-fold more stable complex than Delta or Mu, respectively. Following this, we next extracted *k*_on_ from the BF measured at various contact times by approximating pseudo-first-order kinetics (Figure 2D–F, insets); obtained by varying the duration the tip and surface were in contact.^27,34^ Assuming pseudo-first-order kinetics, the *k*_on_ depends on the effective concentration *c*_eff_, described as the number of binding partners (RBD protein + ACE2 receptor) within an effective volume *V*_eff_ accessible under free equilibrium conditions. We approximated *V*_eff_ by a half-sphere with a radius including the linker, RBD protein, and ACE2 receptor.^22^ For the three biomolecular pairs, we saw an exponential increase in the BF with increasing contact time and found that extracted *k*_on_ values are similar to those retrieved RBD^WT^ and early VoCs.^27^

The extraction of both *k*_on_ and *k*_off_ enables the comparison of the stability of the complexes through their *K*_D_ (ratio of *k*_off_/*k*_on_) Collectively, we obtained *K*_D_ values in the descending order: Mu > Delta > Omicron suggesting altered affinities for the ACE2 receptor. All these values correspond to high-affinity interactions, reminiscent of single-molecule virus-receptor bonds reported previously.^35−38^ In particular, the low *K*_D_ for the Omicron variant (2-fold lower than the RBD^WT^, ∼134 nM) confirms high interface stability due to the synergistic effect of mutations at residues 493, 496, 498, and 501.

### Atomistic Analysis of the Mechanical and Energetic Stability of the RBD/ACE2 Interface

**Figure 3:**
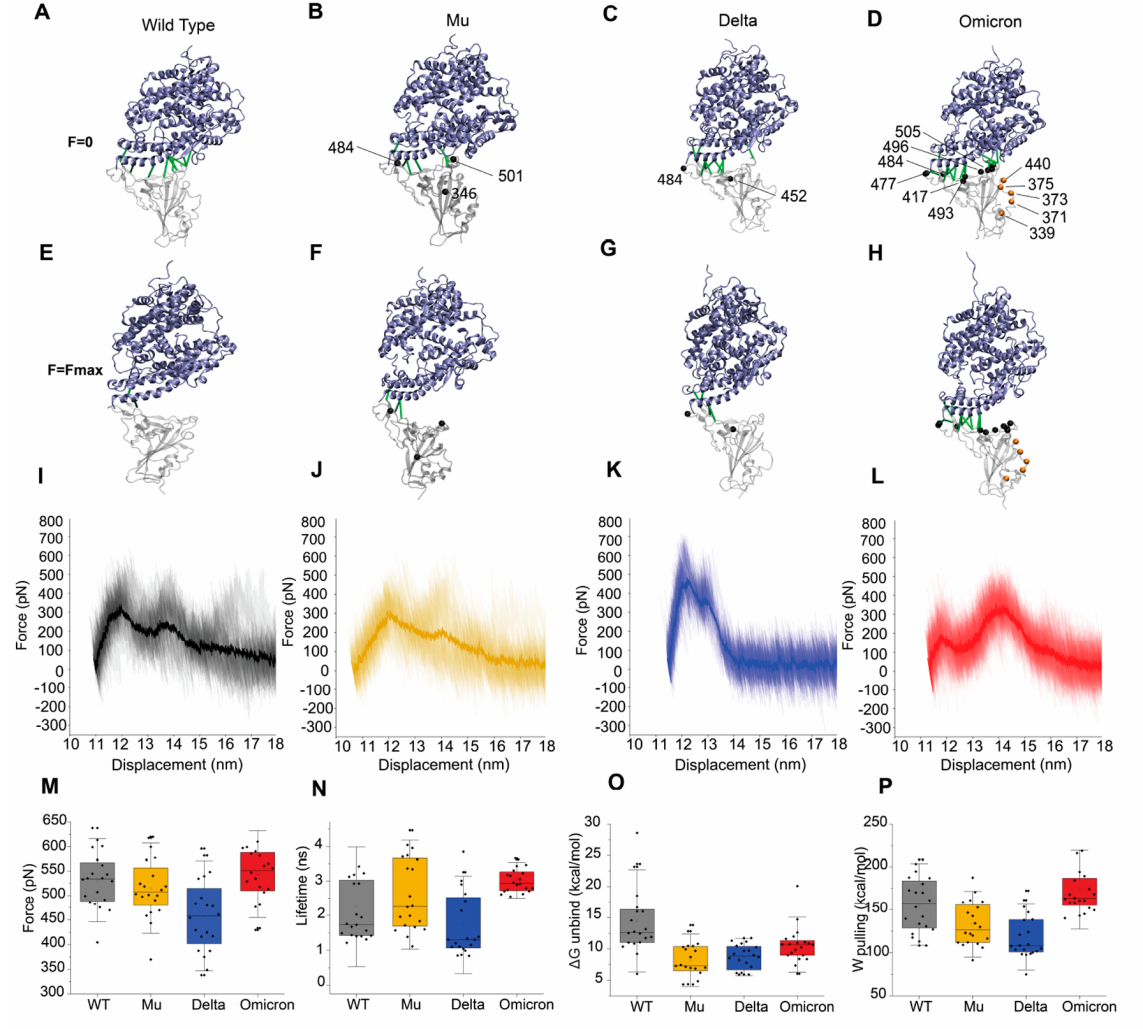
SMD simulation of the RBD/ACE2 complex for WT and three main SARS-CoV-2 variants. Panels showing the ribbon-like representation of the protein complex for WT (A), Mu (B), Delta (C), and Omicron (D). For each variant, the position of the mutated residue is indicated by a solid line. In the case of Omicron, the mutations at the RBD/ACE2 interface are highlighted by black beads and the remaining mutations, which are associated with immune evasion, are shown by orange beads. In addition, we display the interface contacts (green solid line) which are responsible for the mechanical stability and offer a resistance to the detachment of the RBD from the tethered ACE2. (E–H) Snapshots at *F*_max_ in SMD simulations for the WT (E) and the three variants (F–H). Solid green lines represent those contacts still present 50 ps after reaching *F*_max._ (I–L) Cumulative force–displacement graphs for all 20 trajectories of WT, Mu, Delta, and Omicron, respectively. The bold line represents the statistical mean of 20 trajectories. The initial distance value corresponds to the distance between ASP615 and THR333 in ACE2 and RBD, respectively, after the MD equilibration step. (M) External forces (pN) required for the mechanical dissociation of the RBD/ACE2 interface. (N) Lifetime shows the duration of time (in ns) the protein complex remains bound before reaching *F*_max_. (O) The unbinding free energy (Δ*G*_unbind_) is computed by the Jarzynski equality for each system. (P) Work done (W_pulling_) in pulling apart the VoCs from the ACE2 receptor. Data is representative of 20 trajectories and shown as box-whisker plots, wherein each data point belongs to a single trajectory. The square in the box represents the mean, the min/max of the box the 25th and 75th percentiles, respectively, and the whiskers represent the s.d. of the mean value. Middle panels display the RMSD for all cases.

In order to get insightful information behind mechanical stability exhibited by the RBD/ACE2 complexes, we performed SMD simulations mimicking the AFM experiments (Figure 1D). In our simulations, the RBD/ACE2 complex was restrained at position ASP615 (ACE2) and was pulled at a constant velocity via a dummy atom (Cα of THR333 of RBD) until the interface dissociation was observed. These simulations provide a detailed view of the unbinding process and allow for quantification of rupture force, defined as the maximum force (*F*_max_ in pN) reached during the single pulling process (see Figures S5–S7). Using contact map (CM) analysis, we identified contacts present at *F*_max_ and searched for those which vanished after 50 ps, corresponding to a displacement of ≈0.5 Å (see Supplementary Movies 1–4). These events are attributed to stretching of the interaction length of RBD/ACE2 contacts beyond their equilibrium value resulting in bond rupture, suggesting their contribution to *F*_max_ is non-negligible, as depicted by the 2D network representation (Figure S5). To highlight these changes, here we include some snapshots of the initial RBD/ACE2 complex structures (Figure 3A–D), as well as after their subsequent rupture (Figures 3E–H, S5, and S6). It is clear from these snapshots that the interactions present at the interface influence the observed rupture force per trajectory (Figure 3I–L) and are notably different between variants. The number of contacts that vanished was largest for the Omicron variant with 19 contacts, whereas WT and Delta lost 11 and 12 contacts, respectively, and Mu only 6 contacts (see Tables S1–S4). Our analyses also revealed a certain degree of plasticity of the Omicron contacts at *F*_max_ with respect to those present at F = 0 (bound state). We found that ∼90% of contacts displayed by the Omicron RBD under loading were stretched and during SMD simulation vanished abruptly only after reaching *F*_max_. The same analysis for WT, Mu and Delta, shows lower plasticity of the interface where the majority of contacts were vanished before they reached *F*_max_. Our study also revealed that the Omicron/ACE2 complex shows a larger number of stabilizing interactions distributed over the interface, conferring enhanced stability compared to other variants (Figure 3E–H). Additionally, the dissociation resembles an unraveling mechanism due to the more uniform distribution of contacts, as has been reported for other complexes.^39^ Interestingly, we find that for all complexes, the dissociation process starts on the side of RBD residue 501, progressively extending along the interface toward the opposite side (see Supplementary Movies 1–4). For the Mu and Delta variants, which possess more contacts than WT at the opposite end (around RBD residue 484), the dissociation process occurs abruptly in one step.

To quantify mechanostability associated with each variant, we computed the average force ⟨*F*⟩ versus displacement profile according to the SMD trajectories (Figures 3I–L and S7). The average profile reports the following ⟨*F*_max_⟩: 361 ± 99 pN for WT, 331 ± 95 pN for Mu, 509 ± 100 pN for Delta, and Omicron presents two peaks, one centered at 207 ± 54 pN and a second at 379 ± 73 pN. In addition, we report the average *F*_max_ (i.e., ⟨*F*_max_⟩) which is associated with the mechanical stability analysis of contact maps and computed in each of the rupture profiles (Figure 3M): 531 ± 56 pN for WT, 515 ± 61 pN for Mu, 459 ± 75 pN for Delta, and 544 ± 59 pN for Omicron. Analysis of ⟨*F*_max_⟩ shows that the WT and Mu variants have, on average, more peaks at different positions during the dissociation process, whereas Delta and Omicron show a consistent two force peaks in the average rupture profile. The mechanical forces involved in the dissociation process are in the range of 200–600 pN for all variants. The analysis of the lifetimes of the variants shows that Omicron has smaller fluctuations and a large mean value, as it mainly shows two simultaneous peaks in the SMD trajectories in our simulation. This means that the dissociation process follows almost the same path, first passing through a peak (low force) and then a second peak (high force). This second peak is the one that leads to the final dissociation. As a result, this pathway induces less fluctuations in the lifetimes (Figure 3M) of Omicron, whereas the dissociation process in the other variants (i.e., WT, Mu and Delta) also involves two peaks, but this process occurs through either the first or the second peak, resulting in a large dispersion in the lifetimes.

We also computed the nonequilibrium free energy Δ*G*_unbind_ via the Jarzynski inequality, which is the difference between the free energy at the transition state (G^TS^) and *G*_bound_ at the bound state (Figure 3O). We observe that the WT has the highest Δ*G*_unbind_ of 14.5 kcal/mol, and a trend in Δ*G*_unbind_: Omicron > Delta > Mu, which supports stronger ACE2 recognition by the Omicron. Additionally, SMD allowed us to compute the energy required throughout the entire process, referred to as work done (⟨*W*⟩) and found the following ⟨*W*⟩ values for each variant: 153 ± 27 kcal/mol (WT), 120 ± 20 kcal/mol (Mu), 118 ± 20 kcal/mol (Delta), and 174 ± 30 kcal/mol (Omicron) (Figure 3P). Taken together, our SMD results show that Omicron is capable of distributing the mechanical force over a larger contact area thereby increasing its affinity for ACE2, which may be the underlying mechanism behind previous report (Figures S7 and S8).^40^ Having identified the underlying mechanics behind how mutations influence VoC binding behaviors with their target receptor, we next examined their role in antigen recognition.

### Monitoring Immune Neutralization of VoCs

**Figure 4:**
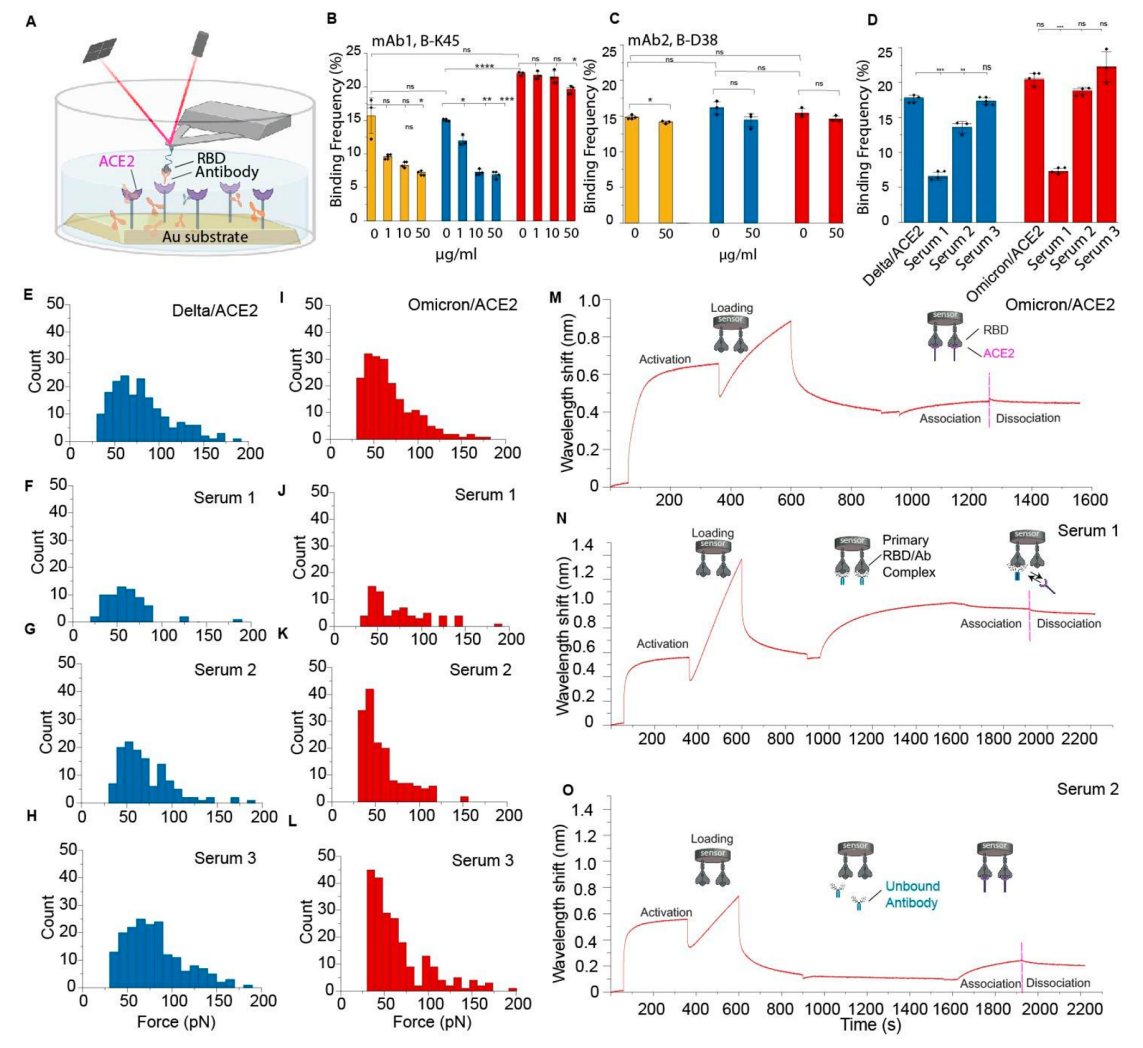
Blocking by monoclonal antibodies (mAbs) and sera from convalescent patients to probe neutralization potential. (A) AFM setup to measure BF of the interaction between ACE2 and the RBD mutants. (B, C) Graph showing blocking potential of mAb 1 and 2 against RBD. Binding frequencies were estimated before and after incubation with mAbs at increasing concentration (1–50 μg mL^–1^ in PBS). Data are representative of at least 3 independent experiments (tips and sample) per mAb concentration. *P*-values were determined by two-sample *t* test in Origin. The error bars indicate SD of the mean value. (D) Graph showing blocking in the presence of Sera 1–2 obtained from convalescent patients and Sera 3 obtained from nonvaccinated and noninfected individual. (E–L) Histogram showing distribution of rupture forces for Delta/ACE2 (in blue) and Omicron/ACE2 (in red) interaction in the presence of Sera 1–3. (*N* = 1024 data points were used to construct each histogram). (M–O) Complete biolayer interferometry (BLI) sensograms highlighting the association and dissociation regime of the Omicron/ACE2 complex in the absence of any sera (M) in the presence of Serum 1 (N) and Serum 2 (O). Experiments in the presence of Sera 1 and 2 were performed after diluting them 1:1000 v/v with 0.1% BSA in PBS.

RBD domains play a critical role in the early stages of infection, controlling the binding of the virus to its host receptor and its ability to infect.^41,42^ To evaluate whether variants could escape host-immune surveillance, we tested a mAb directed against the WT RBD (B-K4) (Figure 4A). In our previous study, we showed that B-R41 only neutralizes the WT and Alpha variant, while B-K45 remained active against the WT, Alpha, Beta, Gamma, and Kappa variants.^27^ Therefore, we decided to further investigate the neutralizing capacity of B-K45 on the Mu, Delta, and Omicron VoCs to determine whether they had acquired new immune evasion properties. To this end, we measured the BF between the respective RBDs and the ACE2 receptor, first in the absence of mAb and after the injection of B-K45 (Figure 4B). We observed that, even though B-K45 was able to significantly reduce the BF by ∼50% for both Delta and Mu variants (IC_50_ at ∼10 μg mL^–1^ for Delta and ∼50 μg mL^–1^ for Mu), its potential is significantly reduced in comparison with previous VoCs.^27^ This is even more clear for the Omicron variant, wherein the B-K45 neutralizing capacity is almost abolished, probably due to the 15 mutations on the RBD surface that synergistically enhance the number of stabilizing contacts. To confirm the specificity of mAb blocking, we also performed a control experiment in the presence of isotype mAb (B-D38) and found no reduction in BF for all three variants, respectively (Figure 4C).

Studies have reported that COVID-19 vaccination increases plasma antibody concentration, with IgG titers increasing 30 times in comparison to nonvaccinated individuals.^6,42,43^ To study the molecular basis of neutralization in a more physiological context, we selected sera from patients who were (i) vaccinated and infected with the Delta variant after vaccination (Serum 1), (ii) nonvaccinated but infected with the Delta variant (Serum 2), and (iii) serum obtained in 2019 from an individual whom had never been in contact with the virus or the vaccine (Serum 3). Using AFM, we evaluated the blocking capacity of these sera on the interaction established between ACE2 and either the Delta or Omicron RBD. We found that while Serum 1 is able to retain its neutralizing potency against both Omicron and Delta variants as shown in Figure 4D (reduction in BF > 50% in both cases), the blocking potency of Serum 2 was significantly reduced (<20%). While Sera 1 and 2 were able to neutralize the RBD/ACE2 interface, experiments performed in the presence of Serum 3, which is devoid of specific antibodies against SARS-CoV-2, showed the complete absence of neutralizing activity (Figure 4D). Furthermore, we constructed force histograms for the blocking experiment with Sera 1–3 and compared them with the histogram obtained in the absence of antibodies (Figure 4E–L). We observed that Sera 1 and 2 are able to reduce the interaction of spike protein with ACE2 as a lower BF is observed for both serum with a lesser extent formed multiple interaction between the RBD-functionalized tip and the ACE 2 surface. In the control serum, complex formation is not affected, as suggested by a very similar force distribution in the histograms (Figure 4H, L). These results underscore the ability of AFM to evaluate the neutralizing power of antibodies, either purified or present in more complex media such as patient serum.

Due to the results of the prior analyses showing its improved binding and mechanical stability, we next used biolayer interferometry (BLI), to monitor the neutralization breadth of antibodies produced by immune responses against the Omicron variant specifically (Figure 4M–O).^44,45^ We measured the avidity between ACE2 and Omicron RBD in the presence of Sera 1 and 2 obtained from convalescent patients. The covalently immobilized Omicron RBD showed a high avidity (*K*_D_ ∼ 9 nM) toward the ACE2 receptors (Figure 4M), with overall higher (∼8 times) *K*_D_ values compared to AFM being attributable to an overestimation of *k*_on_ rates due to rebinding of protein, a caveat associated with bulk measurements. To test the RBD blocking potential of individual serum, experiments were performed in which the biosensor was first loaded with the RBD protein and then incubated with serum to form a primary RBD-antibody (RBD-Ab) complex (Figure 4N,O). This primary RBD-Ab complex was made to react with ACE2 receptors, to further evaluate ability of serum antibodies to interfere with ACE2 binding. In the presence of the intermediate blocking step with Serum 1, the avidity of the ACE2 receptor was almost abolished as shown in the association phase (Figure 4N), presumably due to efficient blocking by the antibodies present, which is in good agreement with our AFM blocking experiments. However, for Serum 2, the antibody binding signal for the RBD domain is strongly reduced, resulting in a lower shift, this weak antibody binding being significantly less effective in blocking ACE2 receptor binding (Figure 4O). Our BLI sensograms along with AFM data highlight the fact that the immune response from an individual post vaccination and/or infection lead to the production of specific antibodies which compete with the virus-receptor recognition step to protect against viral infection.

## Conclusion

Understanding the molecular mechanism of binding for the most recent SARS-CoV-2 VoCs is critical in developing effective treatment strategies and evaluating previous established therapeutics (vaccines and antibodies).^46^ Mutations in the spike protein can alter the protein’s conformation and change the interaction between the virus and host cell receptors, therefore, understanding the binding mechanism of new variants is important to address in the constantly changing landscape of the pandemic

In this study, we combined SMFS and SMD experiments to analyze the stability of RBD/ACE2 complexes, as established by the most recent SARS-CoV-2 VoCs. The extracted kinetic and thermodynamic parameters suggest that the Omicron variant of SARS-CoV-2 forms the most stable complex with the ACE2 receptor guided by a denser network of interactions distributed homogeneously across the interface.^47^

Our results are in good agreement with other studies reporting that Omicron has the ability to evade neutralization by the immune system due to the improved fit between the TYR side chains and a favorable π–π stacking interaction.^16,47,48^ These drastic changes in the interface cause an antigenic shift, resulting in a reduction of the potency of previously developed mAbs like sotrovimab and cilgavimab/tixagevimab.^7,47−49^ Our results reveal a concerning pattern of immune evasion and emphasize the need for continued vigilance and research in monitoring the evolution of this virus and the potential impact on vaccine effectiveness.

## Supporting Information Available

Materials and methods, distribution of forces between Omicron-RBD binding to ACE2, probing Mu-RBD binding to ACE2, probing Delta-RBD binding to ACE2, probing Omicron-RBD binding to ACE2, list of the interface contact for WT and variants, network representation of the most relevant interactions, SMD snapshots capturing the dissociation process, RMSD profiles for all VoCs, RBD/ACE2 interface area (PDF)
Movie of WT: MD trajectory for the dissociation process of the RBD/ACE2 complex described in ribbon-like representation in SMD simulation at pulling speed of 10 Å/ns (MPG)
Movie of Delta: MD trajectory for the dissociation process of the RBD/ACE2 complex described in ribbon-like representation in SMD simulation at pulling speed of 10 Å/ns (MPG)
Movie of Mu: MD trajectory for the dissociation process of the RBD/ACE2 complex described in ribbon-like representation in SMD simulation at pulling speed of 10 Å/ns (MPG)
Movie of Omicron: MD trajectory for the dissociation process of the RBD/ACE2 complex described in ribbon-like representation in SMD simulation at pulling speed of 10 Å/ns (MPG)

## Supplementary Material

ng3c00060_si_001.pdf

ng3c00060_si_002.mpg

ng3c00060_si_003.mpg

ng3c00060_si_004.mpg

ng3c00060_si_005.mpg

## Abbreviations

AFM: atomic force microscopy
DFS: dynamic force spectroscopy
FD: force–distance
BF: binding frequency
SMD: steered molecular dynamics
CM: contact map
mAb: monoclonal antibody
BLI: biolayer interferometry
