# Supplementary material for: Single-Molecule Investigation of the Binding Interface Stability of SARS-CoV-2 Variants with ACE2
Source: ACS Nanosci Au. 2024 Mar 8;4(2):136–45. doi: 10.1021/acsnanoscienceau.3c00060 (PMC11027127; doi:10.1021/acsnanoscienceau.3c00060)
Supplement: Supplementary file 1 — ng3c00060_si_001.pdf [file ng3c00060_si_001.pdf]

Supplementary Information for

Single-molecule investigation of the binding interface stability of SARS-CoV-2 variants with ACE2

Ankita Ray,[1] Thu Thi Minh Tran,[2,3] Rita dos Santos Natividade,[1] Rodrigo A. Moreira,[4] Joshua D. Simpson,[1] Danahe Mohammed,[1, #, ##] Melanie Koehler, [1, ‡] Simon J. L Petitjean,[1] Qingrong Zhang,[1] Fabrice Bureau,[5] Laurent Gillet,[6] Adolfo B. Poma,[7,*] David Alsteens[1,8*]

[1]Louvain Institute of Biomolecular Science and Technology, Université catholique de Louvain, 1348 Louvain-la-Neuve, Belgium
[2] Faculty of Materials Science and Technology, University of Science—VNU HCM, 227 Nguyen Van Cu Street, District 5, 700000 Ho Chi Minh City, Vietnam
[3] Vietnam National University, 700000 Ho Chi Minh City, Vietnam
[4] Basque Center for Applied Mathematics, Mazarredo 14, 48009 Bilbao, Spain
[5] Laboratory of Cellular and Molecular Immunology, GIGA Institute, Liège University, 4000 Liège, Belgium
[6]Immunology-Vaccinology Lab of the Faculty of Veterinary Medicine, Liège University, 4000 Liège, Belgium
[7] Institute of Fundamental Technological Research, Polish Academy of Sciences, Pawińskiego 5B, 02-106 Warsaw, Poland
[8] WELBIO department, WEL Research Institute, 1300 Wavre, Belgium

This PDF file includes:

Materials and methods

- Functionalization of AFM tips
- Preparation of Au model surfaces grafted with ACE2
- FD-based AFM on model surfaces
- Deglycosylation
- All-atom MD simulation
- Steered molecular dynamics (SMD) simulation
- Contact map analysis
- AFM based antibody inhibition assays
- Biolayer interferometry

Tables S1-4

Supplementary Figures S1 to S8

Movies captions

References

Materials and methods

Functionalization of AFM tips. MSCT-D cantilevers (Bruker) were used to probe the interaction between S1 RBD subunits (Diaclone SAS, 715-H26-0BU, 715-H28-0BU, 715-H25-0BU) and extracellular domain of purified ACE-2 receptors (Diaclone SAS, 715-H19-0BU). The cantilevers (MSCT, Bruker, Santa Barbara, USA) were cleaned by immersing in chloroform for 10 min and further cleaned by UV-radiation and ozone. Silanization was performed under an argon atmosphere by placing the clean AFM tips in a desiccator with separate vials containing 3-aminopropylyriethoxysilane APTES (30 µl) and triethylamine TEA (10 µl). The tips were left in the desiccator for 2 hours, following which the desiccator was flushed with argon and the tips were left to cure for two days. Heterobifunctional NHS-PEG_{24}-Ph-aldehyde linkers were used to chemically functionalize APTES coated AFM tips as previously described.[23] Briefly, silanized AFM tips were immersed in a solution of NHS-PEG_{24}-Ph-aldehyde (3.3 mg in 0.5 mL of dry chloroform) in the presence of triethylamine (30 µl). After 2 hours of incubation at room temperature, the cantilevers were thoroughly washed with chloroform three times to remove physiosorbed molecules and dried under a gentle stream of nitrogen.

For AFM tips functionalized with RBD proteins (Delta and Mu RBD) containing a His-tag, 100 µl of a 100 µM tris-nitrilotriacetic amine (tris-NTA) (Toronto Research Chemicals, Canada) was placed on Parafilm, and 2 µl of freshly prepared sodium cyanoborohydride ($NaCNBH_3$) solution was added and thoroughly mixed. Tips were incubated at room temperature in the solution for 2 h. Then, 5 µl of 1 M ethanolamine solution (pH 8.0) was added and incubated for 10 min. The mixture of 50 µl of RBD solution (0.1 mg ml^{-1} in PBS, pH 7.4, and 2.5 µl of 5 mM $NiCl_2$ was placed on the cantilevers and incubated for 2 h at room temperature. After incubation, they were washed in PBS solution three times to remove non-specifically adsorbed molecules. Subsequently, the AFM tips were washed three times with PBS buffer and stored in filtered PBS for a maximum of two days at 4° C.

For AFM tips functionalized with Omicron RBD, 50 µL of protein solution (0.1 mg/ml in PBS buffer pH 7.4) was added to the Ald-PEG_{24}Ph-coated cantilevers placed Parafilm inside a Petridish. To this, sodium cyanoborohydride ($NaCNBH_3$, 1 µL of 1 M stock solution) was added and mixed thoroughly. The tips were incubated for 1 hour at room temperature. Then, ethanolamine hydrochloride (2.5 µL of 1 M stock solution, pH 8.0) was added to block the unreacted aldehyde groups on the AFM cantilever. Subsequently, the AFM tips were washed three times with PBS buffer and stored in filtered PBS for a maximum of two days at 4° C.

Preparation of Au model surfaces grafted with ACE2. Purified hACE2 protein (Diaclone, SAS, 715-H19-0BU) was immobilized on gold-coated surfaces (Au on polished Si wafers) using NHS-EDC chemistry as previously described. Briefly, gold-coated surfaces were rinsed with ethanol, dried under nitrogen, cleaned for 15 min in an UV-ozone cleaner and incubated overnight in solution containing alkanethiol (99% 11-mercapto-1-undecanol 1 mM (Sigma Aldrich) and 1% 16-mercaptohexadecanoic acid 1 mM (Sigma Aldrich) in ethanol). Resultant surfaces were washed with ethanol, dried under a gentle stream of nitrogen, and immersed in a solution 25 mg mL^{-1} mg of dimethylaminopropyl carbodiimide (EDC) and 10 mg mL^{-1} of N-hydroxy succinimide (NHS) for 1 hour at room temperature. Finally, the chemically activated samples were incubated with ACE2 protein (25 µL, 0.1 mg mL^{-1} in PBS, pH 7.4) on parafilm

for 1 hour at 4° C and washed with PBS buffer. Functionalized surfaces were used on the same day of the experiment.

FD-based AFM on model surfaces. FD-based AFM on model surfaces was performed at room temperature in PBS using functionalized MSCT-D probes (Bruker, nominal spring constant of 0.030 N/m and actual spring constants measured prior to the experiment using thermal tune method). A Bruker Nano8 operated in force-volume (contact) mode in fluid (Nanoscope software v9.1) was used for the determination of kinetic on-rate (measuring the binding frequency for different contact times of 0, 50, 100, 150, 250, 500, and 1000 ms) at room temperature. For each force map, a scan size of 5µm, a set point force of 500 pN, resolution of 32 × 32 pixels and a line frequency of 1 Hz were used. A JPK Force Robot 300 AFM (JPK, Germany) was used to measure the loading rates and interaction forces by DFS analysis (using a constant approach speed of 1 µm/s and variable retraction speeds of 0.1, 0.2, 1, 5, 10, and 20 µm/s). The relationship between the pulling velocity (v) and loading rate (LR) is given as:

$$LR = \frac{\Delta F}{\Delta t} = k_{eff}.v$$

where ΔF/Δt being the applied force over time, and k_{eff} the effective spring constant of the system. To identify peaks corresponding to adhesion events occurring between RBD linked to the PEG spacer and the receptor model surface, the retraction curve before bond rupture was fitted with the worm-like chain model (WLC) for polymer extension. The latter expresses the force-extension (F-x) relationship for semi-flexible polymers and is described by the following equation, with l_P the persistence length, L_c the contour length and k_BT the thermal energy:

$$F = \frac{k_BT}{l_p}(\frac{1}{4\left(1 - \frac{x}{L_c}\right)^2} + \frac{x}{L_c} - 0.25)$$

DFS data were extracted using the JPK Data Analysis software (JPK) and further analyzed using Origin software (OriginLab, version 2019) to fit histograms of rupture force distributions for distinct LR ranges. A nonlinear iterative fitting algorithm (Levenberg-Marquardt) was used with the Bell Evans model to extract kinetic and thermodynamic parameters of the interactions. Those data were fitted and K_D calculated as described previously.[21–23,49] For kinetic on-rate analysis, the binding frequency (fraction of curves showing binding events) was determined at a certain hold time (t) (the time the tip is in contact with the surface). In brief, the relationship between interaction time (τ) and BP is described by the following equation:

$$BP = A \times [1 - exp\left(\frac{-(t - t0)}{t}\right)]$$

where A is the maximum BF and t_0 the lag time. Origin software is used to fit the data and extract τ. In the next step, k_{on} was calculated by the following equation, with r_{eff} the radius of the sphere, n_b the number of binding partners, and N_A the Avogadro constant

$$k_{on} = \frac{\frac{1}{2}.4\pi r_{eff}^3.N_A}{3\eta_b\tau}$$

The effective volume V_{eff} ($4\pi r_{eff}^3$) represents the volume in which the interaction can take place. Least-squares fits of the data to a mono-exponential decay curve (line) provide average kinetic

on-rates (k_{on}) of the interaction. This association rate depends on the effective concentration C_{eff}, described as the number of binding partners (RBD protein + ACE2 receptor) within an effective volume V_{eff} accessible under free-equilibrium conditions. We approximated V_{eff} by a half-sphere with a radius including the linker, RBD protein, and ACE2 receptor.
Force-distance curves showing specific single binding events were sorted based on the shape of the rupture events. Unbinding events are considered as specific if they (*i*) significantly detach from the baseline noise (at least a threefold difference), (*ii*) are located at a distance > 12 nm from the contact point (consistent with the PEG spacer extension) and (*iii*) are consistent with the extension of a protein-based polymer (fitted with the worm-like chain model).
All experiments were reproduced at least three times with independent tips and samples. The error bars indicate S.E. of the mean value.

All-atom MD simulation. The initial structure of the RBD-ACE2 complex was obtained from the PDB file with entry 6M0J which corresponds to wild type (WT) case. Further modeling was carried out to accommodate the mutations in Delta, Mu and Omicron RBD variants. For the case of delta 2 mutations (i.e., L452R and T478K), Mu 3 mutation (i.e., R346K, E484K, N501Y) and Omicron 15 mutations (i.e., G339D, S371L, S373P, S375F, K417N, N440K, G446S, S477N, T478K, E484A, Q493R, G496S, Q498R, N501Y, Y505H, T547K) were modeled and energy minimized by UCSF ChimeraX software.[1] The new structures were solvated in a rectangular box with dimensions equal to 14nm x 14nm x 45nm. Our simulation box was composed of about 867000 atoms, which includes 12510 atoms only for RBD-ACE2 complex atoms, about 285000 water molecules. The whole box was neutralized with Na^+ and Cl^- ions. We have employed periodic boundary conditions (PBC) in all three Cartesian coordinates. To perform MD simulation we employed CHARMM36m[2] force field for proteins and TIP3P model for water molecules. The leapfrog algorithm[3] was used to integrate the equations of motion with a time step of 2 fs. The LINCS algorithm[4] was used for molecular simulation with bond constrains.[4] The velocity of atoms was changed periodically by v-rescale temperature coupling which maintained the temperature stable with a relaxation time of 0.1 ps over the whole MD simulation. Short range cutoff radius of 1.4 nm was used to calculate the van der Waals (vdW) and electrostatic forces. To calculate the long-range electrostatic interaction, we employed the particle mesh Ewald (PME) method. The system temperature was maintained at 300K using the V-rescale thermostat algorithm and the pressure was kept at 1 bar using the Parrinello-Rahman algorithm.[5] MD simulations were conducted using the GROMACS package, version 2020.4.[6]

Deglycosylation

N-glycans removal: The functionalized AFM tip was treated N-Glycosidase enzyme using PNGase F kit from NEB (New England Biolabs). Briefly,_the tip was incubated with 1 µL PNGase enzyme (500 units/µL), 4 µL Rapid PNGase buffer (5x), and 5 µL ddH2O for a total of 20 µL reaction and incubated at 50 °C for 10 min following which the tip was washed thrice with PBS and mounted on the AFM head.

O-glycans removal: O-Glycosidase assay using O-Glycosidase kit from NEB (New England Biolabs): Briefly,_the tip was incubated with incubated with 2 µL O-glycosidase enzyme (40,000 units/µL), 4 µL Glyc buffer (10x), 4 µL NP40 (10%), and 10 µL ddH2O, for a total of 40 µL at 37°C. After this step, which the tip was washed thrice with PBS and mounted on the AFM head.

Steered Molecular Dynamics (SMD) simulation. The molecular system was energy minimized by the steepest descent method and equilibrated by performing an NVT simulation for 1 ns and followed by NPT simulation for another 1 ns. These two steps maintained the temperature and pressure at 300 K and 1.0 bar respectively. The last snapshot of the NPT equilibration was chosen as the initial structure for the SMD simulation.

The RBD/ACE2 complexes were placed in a rectangular box that has a size large enough to accommodate the full detachment of the RBD under a pulling force from the anchored ACE2 receptor and also satisfy the minimum image convention condition. An external force was applied to a dummy atom, which is connected with the Cα atom of the residue THR-333 in the RBD, through a spring with a stiffness k. The pulling direction was chosen parallel to the longest side of the simulation box. To prevent the ACE2 receptor to be dragged along the pulling direction, the position of Cα atom of the residue ASP-615 was restrained.

The force experienced by the pulled atom is F= k(vt –x), where x is its displacement from the initial position and v is a pulling speed. The spring constant k was set to 600 kJ/mol/nm^2 which is the typical value used in the AFM experiment.[20] Like preceding studies[57,58] in all SMD simulations the pulling speed was chosen to be v = 10 Å/ns, corresponding to a loading rate of 999.6 x 10^9 pN/s, which is much higher than typical AFM experimental speed. However, as confirmed by our studies the pulling speed will affect the absolute value of the rupture force and work, but the relative mechanical stability should not depend on v.[59,60] With this choice of k and v parameters, the mechanical stability of protein systems is in qualitative agreement with experiments on protein-ligand, protein-protein and fibril-like systems.

As shown previously, the rupture force F_{max}, can be used to characterize the mechanical stability of the protein complex. In addition, we can use the pulling work (W) to characterize the systems, defined by:

$$W = \int f(x)dx = \frac{1}{2}\sum_{i=1}^{n-1}(f_{i+1} + f)(x_{i+1} - x_i) \qquad (1)$$

where n is the number of simulation steps, f_i and x_i are the force experienced by the pulled chain and position at step i. The pulling work W is more robust than F_{max} because the pulling work is a function of the entire process, while the rupture force is determined only in a single state. Moreover, the mechanical stability in terms of the free energy (ΔG) can be obtained using Jarzynski's equality, as was shown by. by In this regard, Hummer and Szabo, by showed that ΔG is related to W by the following equation,

$$exp\left(\frac{-\Delta G}{k_B T}\right) = \left\langle exp\left(\frac{W(t) - \frac{1}{2}k(z_t - vt)^2}{k_B T}\right)\right\rangle_N \qquad (2)$$

where <…>N stands for averaging over N trajectories, z_t is the displacement of the pulled atom along the pulling direction and W is given by Eq. (1). We performed 20 independent SMD simulations (i.e., N=20). Since z_t is also known from the simulation, then we can calculate exp(-[W-k(z_t-vt)^2/2]/k_BT) for each trajectory and also then calculate the average over the ensemble of trajectories to get ΔG as a function of time (or the related displacement).

Contact map analysis of the RBD/ACE2 interface of variants. The determination of the most relevant protein-protein interactions at the RBD/ACE2 interface follows our contact map analysis which has been already validated in several protein complexes and also for the study of the SARS-CoV-2 spike glycoprotein [16]. The contact maps are based on the OV + rCSU approach and it has been stored in our server http://pomalab.ippt.pan.pl/GoContactMap/). We obtained from our SMD trajectories 500 contact maps which were sampled every 50 ps of simulation time. Then we performed a detailed analysis of contacts which disappeared between the subsequent contact maps. This step allows us to identify the most relevant interactions that lead to F_{max}. Moreover, when we observe a drop in the forces (F) and this effect is correlated with sudden loss of contacts, hence the CM highlights the set of relevant contacts which are contributing the most to the force peak for each trajectory. Finally, we construct out of the whole 20 trajectories a set of the most stable interaction which gives stability to the RBD/ACE2 interface during the SMD simulation.

Study participants. Sera were obtained from the University Hospital of Liège biobank (SARSSURV-ULiège, University Hospital of Liège Ethics Committee, reference number 2021/96.

AFM based antibody inhibition assays. The binding frequency for the interaction between RBD of different mutants and the ACE-2 receptor were measured before and after incubation with different concentrations (1, 10 and 50 μg mL^{-1} in PBS) of two different monoclonal antibodies (B-K45 and B-D38-isotype control) (Diaclone SAS, France). Briefly, three force-volume maps were recorded on three different areas as described previously in the absence of any antibody (i.e., force-volume mode, 1μm/s approach and retraction speed, map size of 5μm, threshold force of 500 pN, 32 × 32 pixels, 512 samples/line, frequency of 1Hz, and contact time of 250 ms on surface). Thereafter, antibodies were added to the fluid cell and three maps were recorded for each concentration.

Biolayer Interferometry. Affinity between the RBD and ACE2 was measured on a BLI instrument (Octet, ForteBio, Sartorius) device equipped with an amine-reactive biosensor (Octet, ForteBio, Sartorius, Göttingen, Germany). The Omicron RBD was grafted via NHS/EDC coupling step followed by a quenching step with ethanolamine.[49] Then the biosensor was dipped in a PBS well to collect a second baseline following which it was dipped in a well containing ACE2 (0.025 mg/ml in PBS, 300 s, association phase). Finally, the dissociation was performed in a well with PBS (300s). All measurements were conducted at 25 °C and shaker speed 1000 rpm. The association and dissociation part of the curve was fit by a Langmuir 1:1 stoichiometric model to obtain the dissociation constant using the Octet Analysis software.

For the blocking experiment, the RBD was grafted by NHS/EDC coupling, and quenched by ethanolamine. After a washing step in PBS, the biosensor was dipped in a well containing a mixture of (1 μl of sera, 4 μl of 1% BSA solution in PBS, and 195 ul of PBS to give a total volume of 200 μl) to form the primary RBD-Ab complex. The biosensor was then dipped in

PBS to remove non-specifically adsorbed antibodies. The association step was performed in ACE2 (0.025 mg/ml in PBS, 300s) followed by the dissociation phase in PBS.

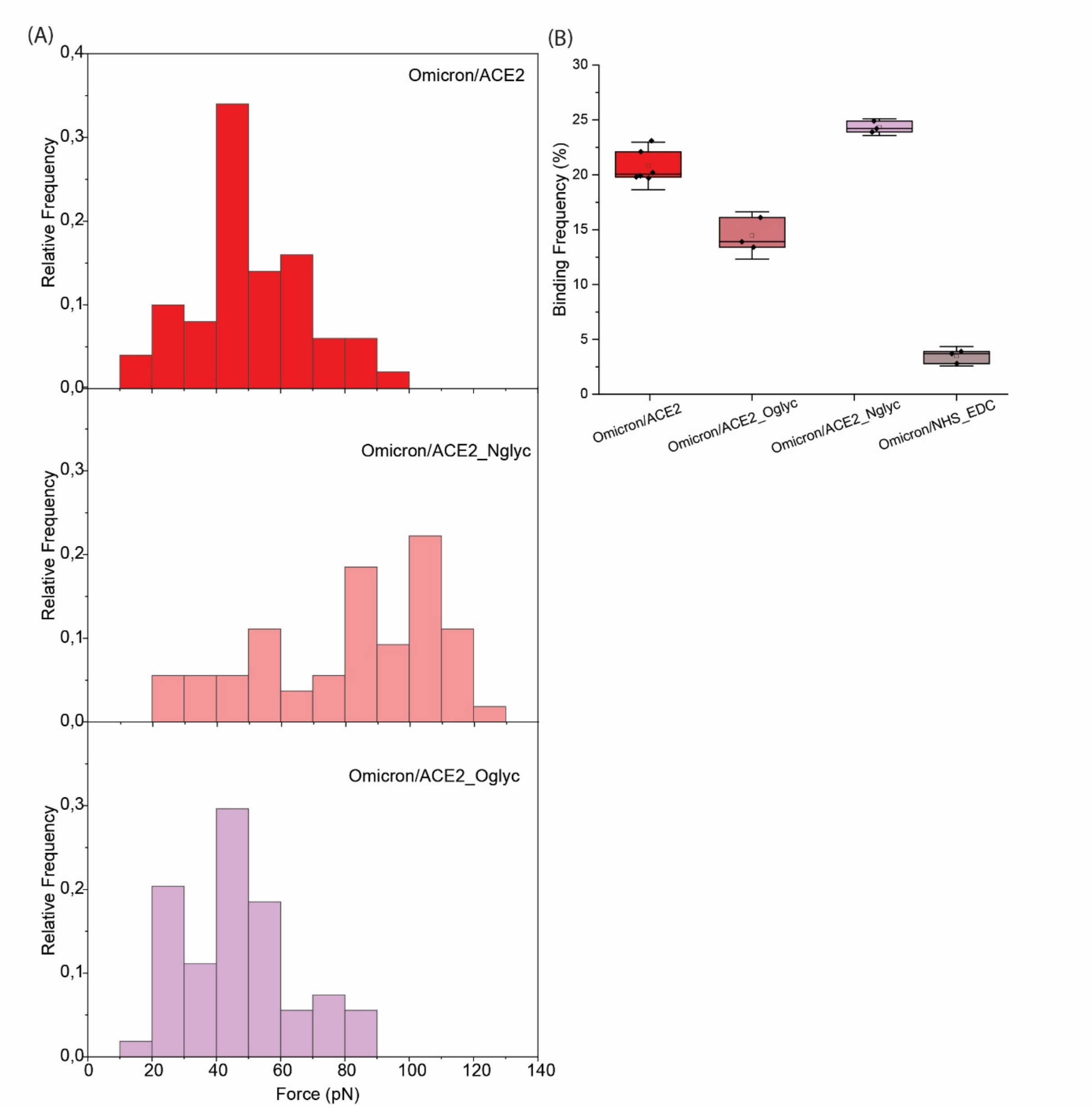

Figure S1. Distribution of forces between Omicron-RBD binding to ACE2 functionalized model surfaces. **(A)** Forces obtained from the interaction at the RBD/AC2 interface (top panel) after removal of N-glycans with N-glycosidase (middle panel) and after removal of O-glycans with O-glycosidase. (bottom panel). Data obtained from 1 map measured by contact mode in fluid consisting of 1024 data points for each condition. Contact time: 0 ms, FV/RV: 1 µm/s. **(B)** Box plot of binding frequencies after enzymatic treatment of the functionalized cantilever with N-glycosidase, O-glycosidase and an NHS/EDC gold surface lacking the ACE2 protein. A data point corresponds to the BF from a map acquired at a retraction speed of 1µm/s. Each data point corresponds to a map with 1024 FD curves. The square in the box represents the mean, the min/max of the box represents the 25th and 75th percentiles, respectively, and the whiskers represent the s.d. of the mean.

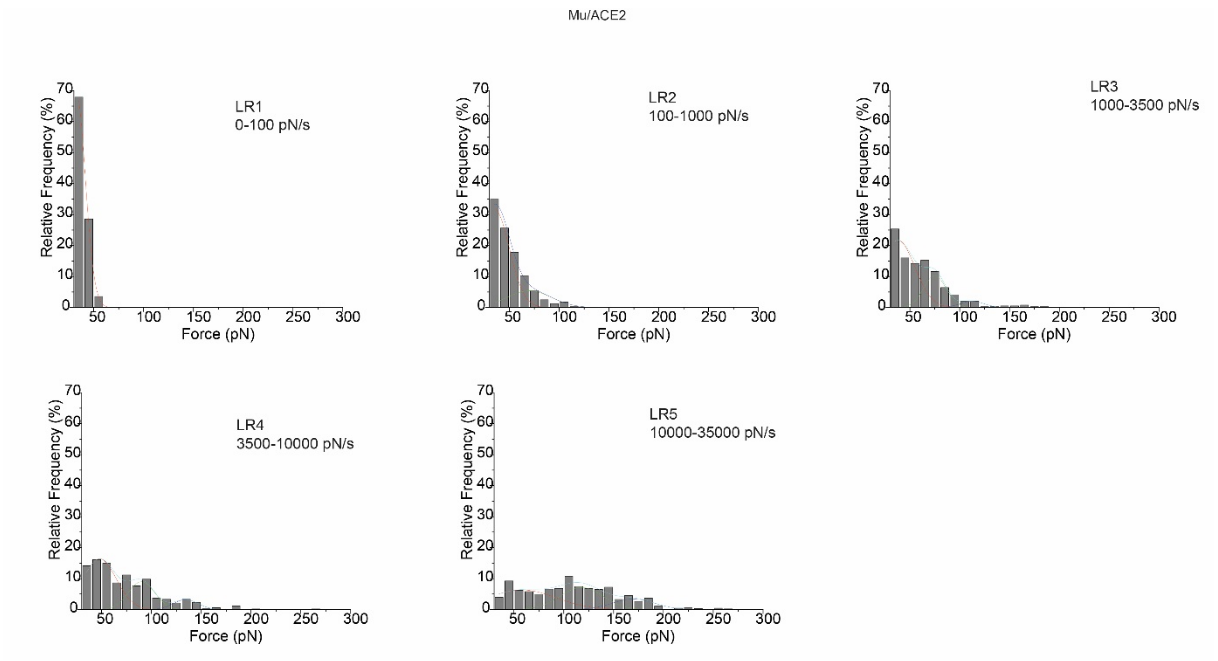

Figure S2. Probing Mu-RBD binding to ACE2 functionalized model surfaces. Force and LR were extracted from force-distance curves and sorted in distinct LR ranges (LR1-LR5). The rupture forces for each LR range were plotted as histograms and fitted with multipeak Gaussian fits. The maxima of all force peaks are indicated. N = 2785 from 6 independent maps for each LR range.

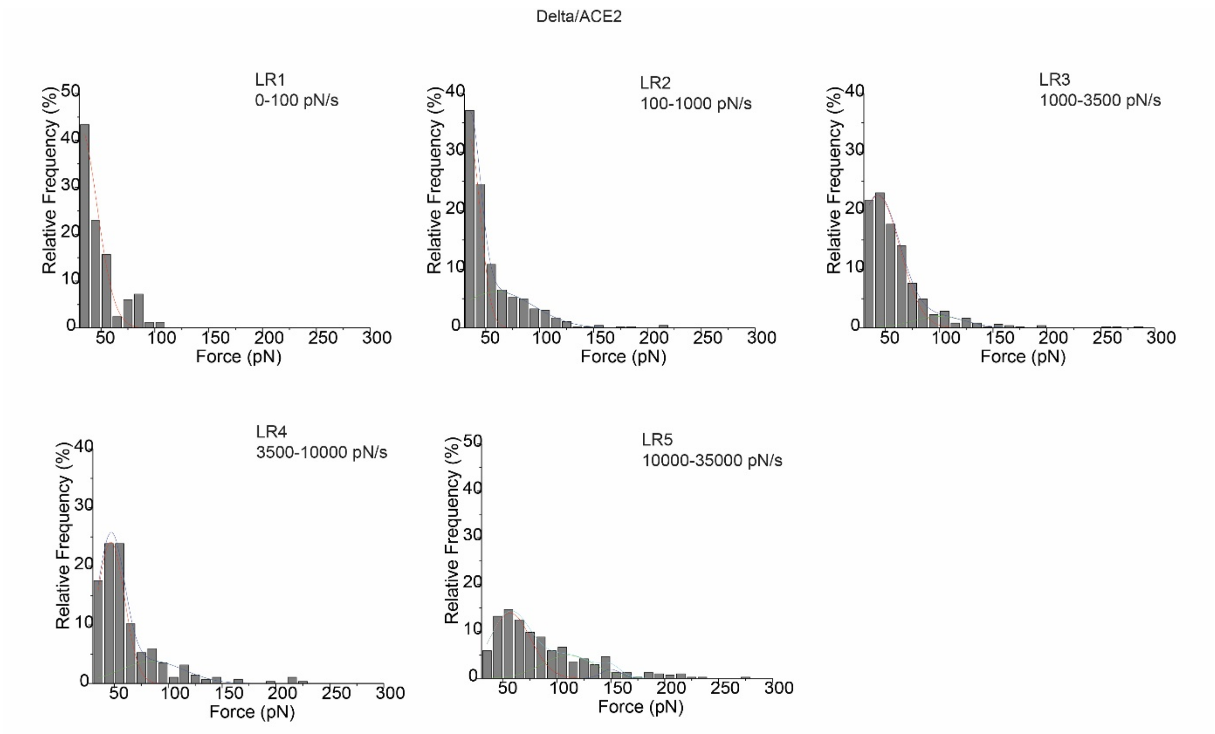

Figure S3. Probing Delta-RBD binding to ACE2 functionalized model surfaces. Force and LR were extracted from force-distance curves and sorted in distinct LR ranges (LR1-LR5). The rupture forces for each LR range were plotted as histograms and fitted with multipeak Gaussian fits. The maxima of all force peaks are indicated. N = 2411 from 6 independent maps for each LR range.

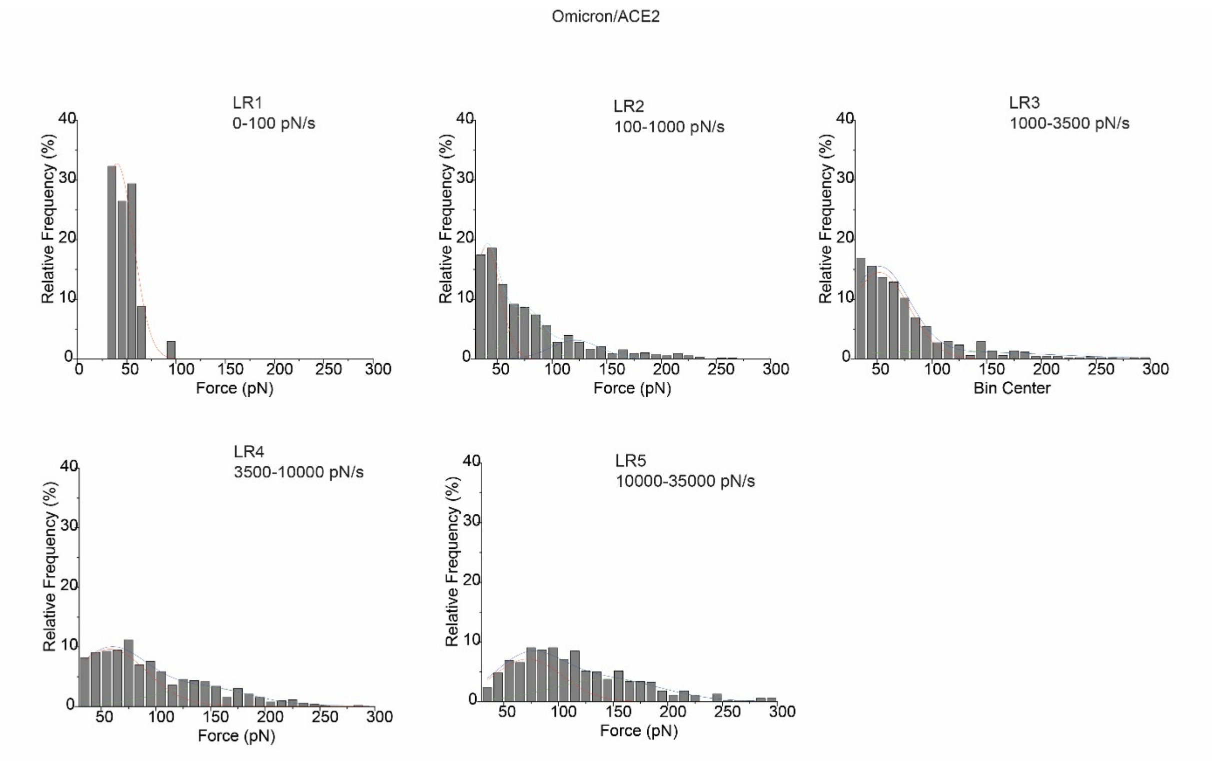

Figure S4. Probing Omicron-RBD binding to ACE2 functionalized model surfaces. Force and LR were extracted from force-distance curves and sorted in distinct LR ranges (LR1-LR5). The rupture forces for each LR range were plotted as histograms and fitted with multipeak Gaussian fits. The maxima of all force peaks are indicated. N = 2698 from 6 independent maps for each LR range.

Table S1: List of the most relevant 11 interface contacts at F_{max} during SMD for WT. Protein-protein interactions are calculated via OV+rCSU contact map protocol. Distance between two $C\alpha$ forming a contact and type of interaction are also reported.

Residue in ACE2	Residue in RBD	d($C\alpha$-$C\alpha$)[Å]	Type of interaction
GLN42	GLN498	10.67	Polar
LYS353	TYR505	9.30	
THR27	TYR489	11.04	
HIS34	GLN493	10.75	
GLN42	TYR449	12.75	
ASP355	THR500	8.51	
ARG357	THR500	12.16	
LEU79	PHE486	8.94	Hydrophobic
TYR41	THR500	11.60	
TYR41	GLN498	10.08	
GLN24	ASN487	9.11	Polar

Table S2: List of the most relevant 12 interface contacts at F_{max} during SMD for Delta variant.

Residue in ACE2	Residue in RBD	d($C\alpha$-$C\alpha$)[Å]	Type of interaction
GLN24	ASN487	9.88	polar
LYS31	GLN493	11.01	
HIS34	LEU455	10.45	
MET82	PHE486	10.24	Hydrophobic
GLU35	GLN493	10.94	
THR27	TYR473	12.93	
THR27	TYR489	10.67	
LYS31	TYR489	9.98	
LEU45	THR500	10.61	
LEU79	PHE486	8.72	Hydrophobic
THR27	PHE456	13.31	
LYS31	PHE456	9.19	

Table S3: List of the most relevant 6 interface contacts at F_{max} during SMD for Mu variant.

Residue in ACE2	Residue in RBD	d(Cα-Cα)[Å]	Type of interaction
THR27	PHE456	11.71	
GLN42	GLN498	10.93	Polar
LEU79	PHE486	9.63	
LYS353	TYR505	9.56	
THR27	TYR489	10.71	
LYS31	GLN493	11.17	

Table S4: List of the most relevant 19 interface contacts at F_{max} during SMD for Omicron variant.

Residue in ACE2	Residue in RBD	d(Cα-Cα)[Å]	Type of interaction
THR27	TYR473	13.33	
LYS31	PHE456	9.78	
LYS31	TYR489	9.64	
TYR41	THR500	13.27	
LYS353	TYR501	11.78	
SER19	ALA475	8.64	
LYS353	GLY502	6.34	
GLY354	GLY502	4.69	
ARG357	THR500	12.57	
MET82	PHE486	9.51	Hydrophobic
GLY354	HIS505	7.28	
SER19	ASN477	6.51	Polar
THR27	TYR489	10.70	
LEU79	PHE486	8.33	Hydrophobic
SER19	GLY476	8.90	
HIS34	LYS493	11.12	
THR27	PHE456	13.62	
HIS34	LEU455	11.33	
GLU35	LYS493	11.04	Ionic

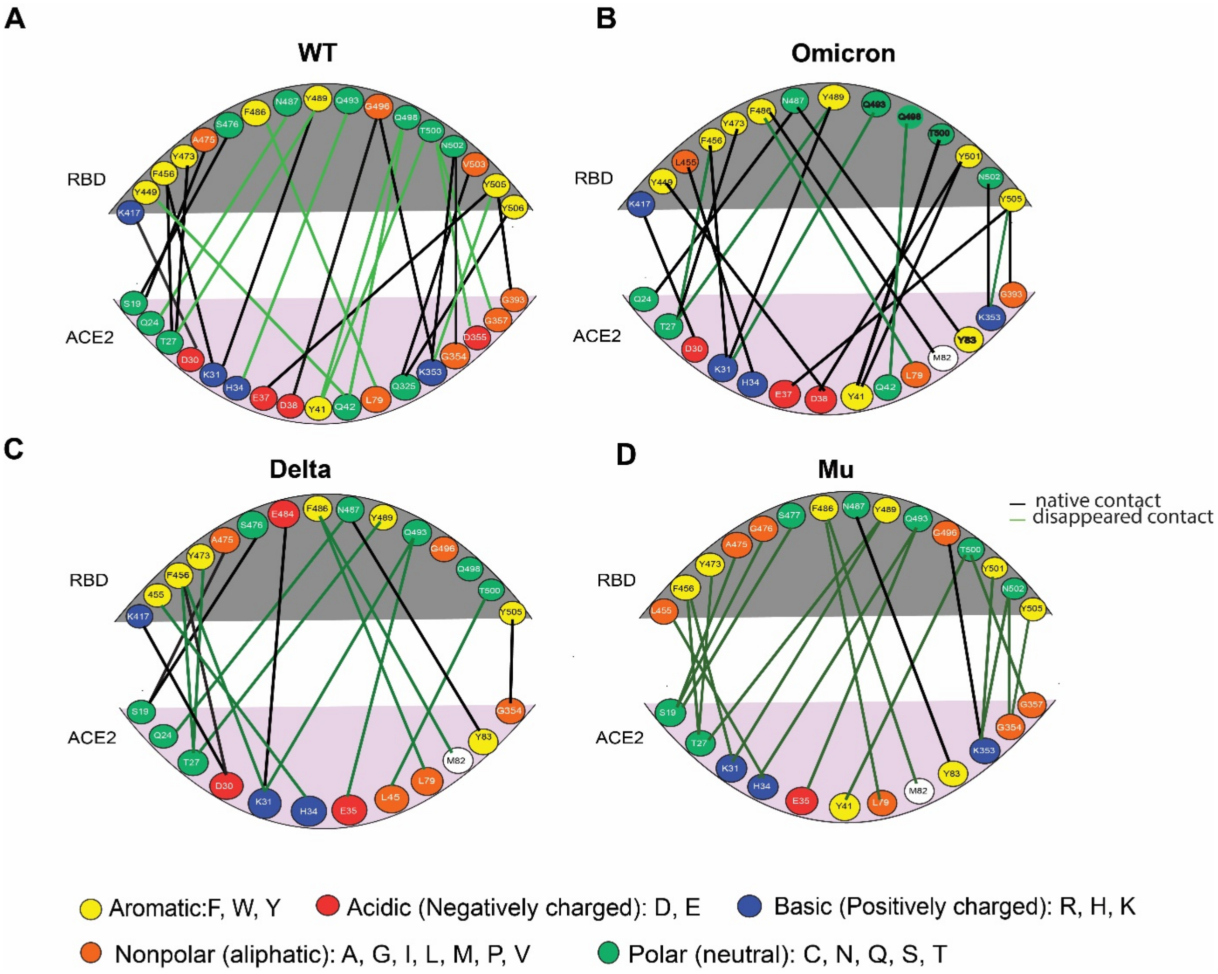

Figure. S5. Network representation of the most relevant interactions or "contacts" present in the RBD/ACE2 interface for several variants. The solid green line represents a contact that is stretched during the SMD simulation and vanishes at F_{max}, whereas solid black line indicates a contact that vanished before F_{max}. Amino acids residues are coloured according to their chemical character (i.e. yellow for aromatic, red for acidic, blue for basic, orange for nonpolar and green for polar).

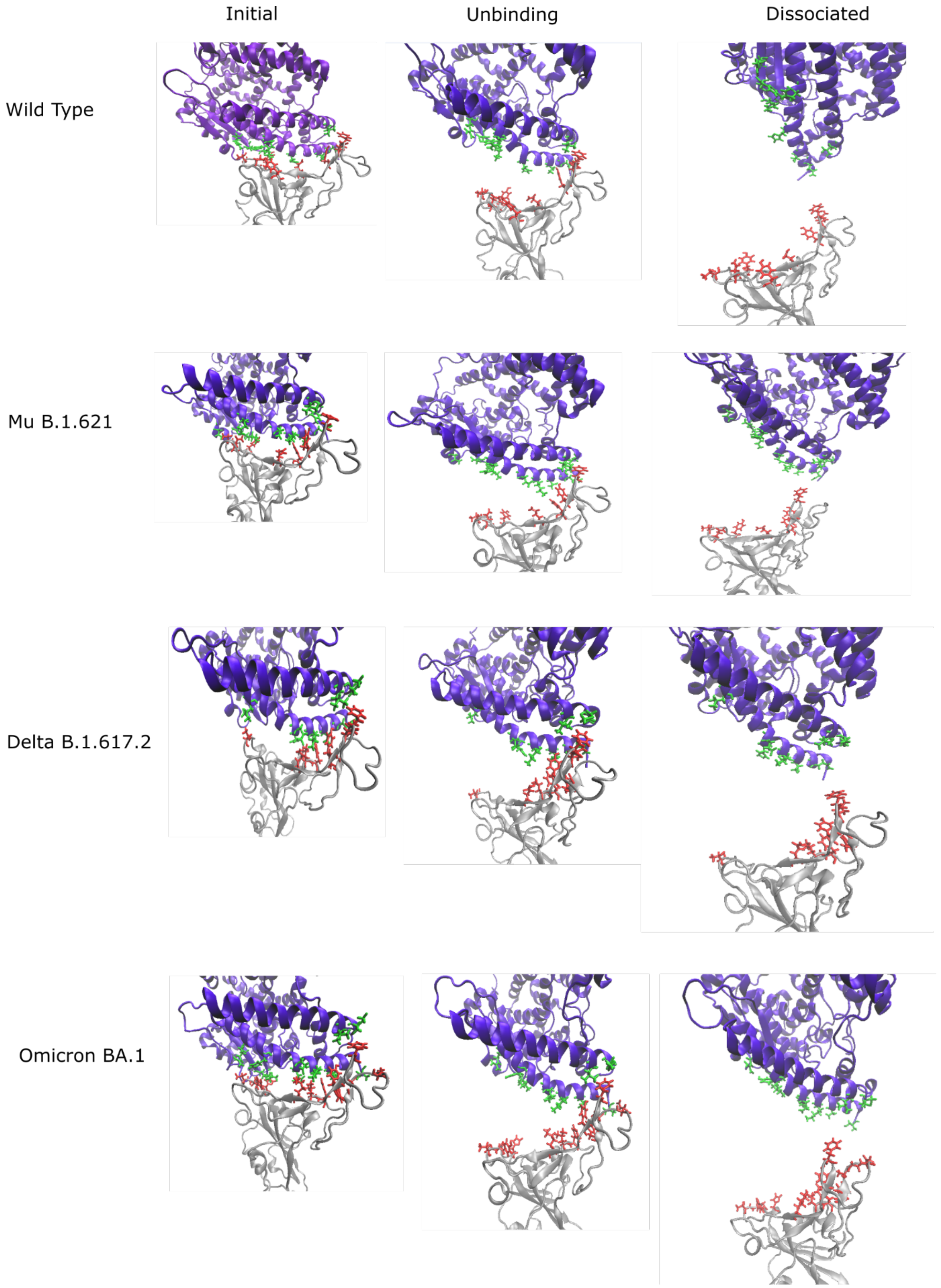

Figure S6. Snapshots capturing the dissociation by steps as well as different conformation at the level of sidechains during the dissociation process. The three panels present 3 clear stages during the dissociation of the RBD from ACE2: bound (left), at F_{max}(middle) and unbound states (right). The sidechains in the picture belong to those residues that are responsible for F_{max}.

We show the effect of the force when it reaches the corresponding ACE2 of Mu and Omicron variants (RMSD ~ 0.4 nm), whereas this effect is smaller in the Delta and WT variant reaching ~0.3 nm. Finally, we show in **Figure S7**, the normalized number of native contacts in ACE2. A deviation from 1 would mean that an internal reorganization of the ACE2 receptor occurs during the application of force by SMD.

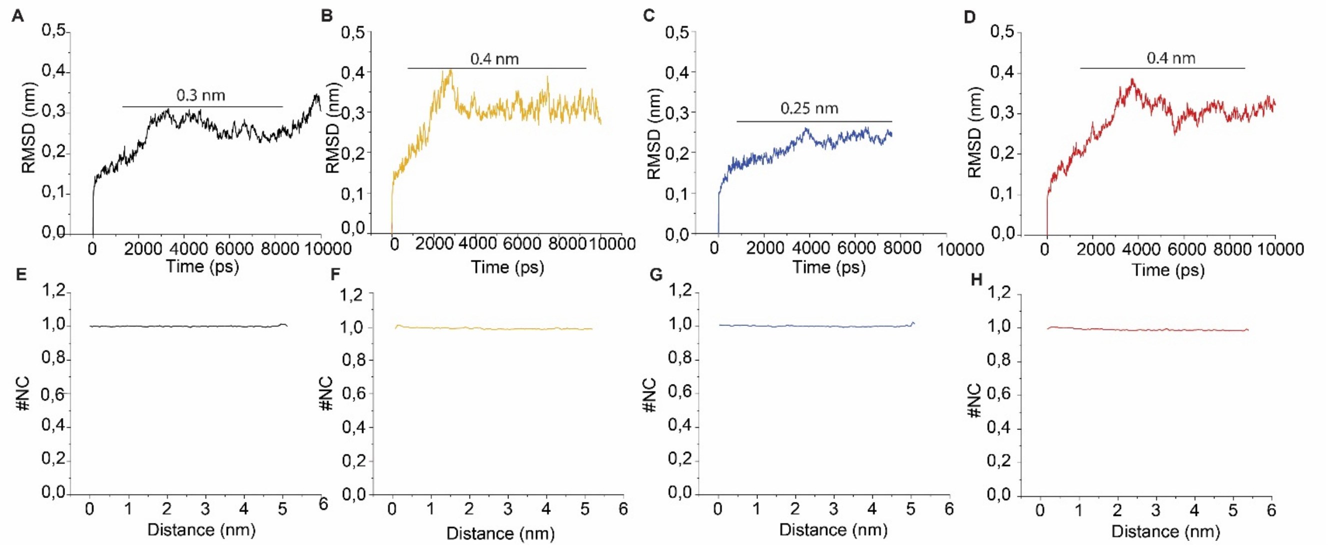

Figure S7. Panels display the RMSD for all cases. Panel (A) shows RMSD profile for WT, (B) for Mu, (C) for Delta and (D) for Omicron. (E-H) Bottom panels show the evolution of the normalized number of contacts during SMD. (#NC=NC(t)/NC(t=0), in the range of 0 to 1) Overall, the observed changes are below ~1% that vanished for Mu and Omicron variants, while for WT and Delta variants the changes are close to initial reference values. These results confirm only minor conformational changes in process. Color convention is as described in Figure 3 in the main text.

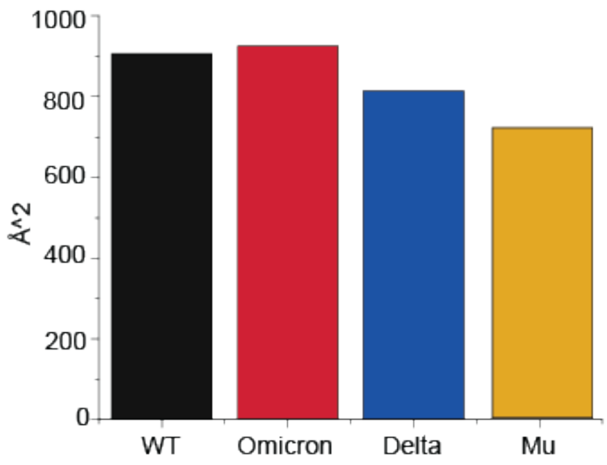

Figure S8. The RBD/ACE2 interface area computed by https://www.ebi.ac.uk/pdbe/pisa/.

Movies captions

Movie of WT: MD trajectory for the dissociation process of the RBD/ACE2 complex described in ribbon-like representation in SMD simulation at pulling speed of 10 Å/ns. The movie has been rendered in VMD software. 11 interface contacts (see Table S1) are highlighted as green solid lines and they are conditioned to disappear if the equilibrium length of each bond increases.

Movie of Delta: MD trajectory for the dissociation process of the RBD/ACE2 complex described in ribbon-like representation in SMD simulation at pulling speed of 10 Å/ns. The movie has been rendered in VMD software. 12 interface contacts (see Table S2) are highlighted as green solid lines and they are conditioned to disappear if the equilibrium length of each bond increases.

Movie of Mu: MD trajectory for the dissociation process of the RBD/ACE2 complex described in ribbon-like representation in SMD simulation at pulling speed of 10 Å/ns. The movie has been rendered in VMD software. 6 interface contacts (see Table S3) are highlighted as green solid lines and they are conditioned to disappear if the equilibrium length of each bond increases.

Movie of Omicron: MD trajectory for the dissociation process of the RBD/ACE2 complex described in ribbon-like representation in SMD simulation at pulling speed of 10 Å/ns. The movie has been rendered in VMD software. 19 interface contacts (see Table S4) are highlighted as green solid lines and they are conditioned to disappear if the equilibrium length of each bond increases.

References:

(1) Pettersen, E. F.; Goddard, T. D.; Huang, C. C.; Meng, E. C.; Couch, G. S.; Croll, T. I.; Morris, J. H.; Ferrin, T. E. UCSF ChimeraX: Structure Visualization for Researchers, Educators, and Developers. *Protein Sci.* **2021**, *30* (1), 70–82. https://doi.org/10.1002/pro.3943.

(2) Huang, J.; Rauscher, S.; Nawrocki, G.; Ran, T.; Feig, M.; De Groot, B. L.; Grubmüller, H.; MacKerell, A. D. CHARMM36m: An Improved Force Field for Folded and Intrinsically Disordered Proteins. *Nat. Methods* **2016**, *14* (1), 71–73. https://doi.org/10.1038/nmeth.4067.
(3) Van Gunsteren, W. F.; Berendsen, H. J. C. A Leap-Frog Algorithm for Stochastic Dynamics. *Mol. Simul.* **1988**, *1* (3), 173–185. https://doi.org/10.1080/08927028808080941.
(4) Hess, B.; Bekker, H.; Berendsen, H. J. C.; Fraaije, J. G. E. M. LINCS: A Linear Constraint Solver for Molecular Simulations. *J. Comput. Chem.* **1997**, *18* (12), 1463–1472. https://doi.org/10.1002/(SICI)1096-987X(199709)18:12<1463::AID-JCC4>3.0.CO;2-H.
(5) Bussi, G.; Donadio, D.; Parrinello, M. Canonical Sampling through Velocity Rescaling. *J. Chem. Phys.* **2007**, *126* (1). https://doi.org/10.1063/1.2408420.
(6) Abraham, M. J.; Murtola, T.; Schulz, R.; Páll, S.; Smith, J. C.; Hess, B.; Lindah, E. Gromacs: High Performance Molecular Simulations through Multi-Level Parallelism from Laptops to Supercomputers. *SoftwareX* **2015**, *1–2*, 19–25. https://doi.org/10.1016/j.softx.2015.06.001.
